# Camel *Streptococcus agalactiae* populations are associated with specific disease complexes and acquired the tetracycline resistance gene *tetM* via a Tn*916*-like element

**DOI:** 10.1186/1297-9716-44-86

**Published:** 2013-10-01

**Authors:** Anne Fischer, Anne Liljander, Heike Kaspar, Cecilia Muriuki, Hans-Henrik Fuxelius, Erik Bongcam-Rudloff, Etienne P de Villiers, Charlotte A Huber, Joachim Frey, Claudia Daubenberger, Richard Bishop, Mario Younan, Joerg Jores

**Affiliations:** 1 International Livestock Research Institute, Old Naivasha Road, PO Box 30709, 00100 Nairobi, Kenya; 2 Molecular Biology and Bioinformatics Unit, International Centre for Insect Physiology and Ecology, P.O. Box 30772, 00100 Nairobi, Kenya; 3 Federal Office of Consumer Protection and Food Safety (BVL), Diedersdorfer Weg 1, 12277 Berlin, Germany; 4 SLU-Global Bioinformatics Centre, Department of Animal Breeding and Genetics, Swedish University of Agricultural Sciences, P.O. Box 7023, SE-750 07 Uppsala, Sweden; 5 Centre for Clinical Vaccinology and Tropical Medicine, Churchill Hospital, University of Oxford, Oxford, United Kingdom; 6 Department of Medical Parasitology and Infection Biology, Swiss TPH, Socinstrasse 57, Basel CH-4002 Switzerland; 7 Institute of Veterinary Bacteriology, University of Bern, Laenggass-Str. 122, CH-3001 Bern, Switzerland; 8 Vétérinaires Sans Frontières Germany, P.O. Box 25653, 00603 Nairobi, Kenya

## Abstract

Camels are the most valuable livestock species in the Horn of Africa and play a pivotal role in the nutritional sustainability for millions of people. Their health status is therefore of utmost importance for the people living in this region. *Streptococcus agalactiae,* a Group B *Streptococcus* (GBS), is an important camel pathogen. Here we present the first epidemiological study based on genetic and phenotypic data from African camel derived GBS. Ninety-two GBS were characterized using multilocus sequence typing (MLST), capsular polysaccharide typing and in vitro antimicrobial susceptibility testing. We analysed the GBS using Bayesian linkage, phylogenetic and minimum spanning tree analyses and compared them with human GBS from East Africa in order to investigate the level of genetic exchange between GBS populations in the region. Camel GBS sequence types (STs) were distinct from other STs reported so far. We mapped specific STs and capsular types to major disease complexes caused by GBS. Widespread resistance (34%) to tetracycline was associated with acquisition of the *tetM* gene that is carried on a Tn*916*-like element, and observed primarily among GBS isolated from mastitis. The presence of *tetM* within different MLST clades suggests acquisition on multiple occasions. Wound infections and mastitis in camels associated with GBS are widespread and should ideally be treated with antimicrobials other than tetracycline in East Africa.

## Introduction

In many semiarid and arid regions of the Horn of Africa, camel keeping is the most sustainable livestock enterprise. Due to climate change and desertification, cattle numbers are decreasing in such regions while camel numbers are increasing and are likely to play an even more significant role for human nutrition in the future [1]. For the people living in these harsh dry areas, the camels play a pivotal role in survival as an important source of animal protein, especially milk and to a lesser extent meat, transportation, cultural status and financial reserve [2]. People live in very close contacts with their animals and camel milk is traditionally consumed raw without proper heat-treatment, which poses a risk for acquiring infections with zoonotic pathogens [3]. The potential risk of transmission of camel pathogens to humans therefore requires investigation.

*Streptococcus agalactiae*, a Group B *Streptococcus* (GBS) is an important pathogen affecting humans and livestock species such as cattle. This pathogen has also been isolated from both healthy and diseased camels from the Horn of Africa [4-9].

The uncontrolled distribution and usage of antibiotics to treat bacterial livestock infections in the Horn of Africa [10] is likely to contribute to the emergence and transmission of antimicrobial resistance genes and requires further investigation [11]. Data on antimicrobial susceptibility in camel GBS in Africa is scanty at best, with only few antibiotics tested on a limited number of samples [5].

*S. agalactiae* possesses a polysaccharide capsule that can be divided into ten different types based on molecular typing [12]. Certain capsular types have been associated with invasive disease or asymptomatic carriage. Capsular type III *Streptococcus* is predominant among the types causing invasive neonatal infections [13,14]. However, capsular type V has also recently emerged as the cause of a significant proportion of invasive human infections in North America [15].

This study aimed to gain insight into the genetic and phenotypic diversity of camel GBS isolates from East Africa in order to guide the development of diagnostic assays as well as vaccines and to provide data useful for informing antimicrobial treatment strategies for control of diseases in camels caused by GBS in the horn of Africa. We characterized 92 camel GBS isolates, their capsular types, tested their antibiotic resistance profile and the resistance genes. Additionally, we typed the isolates by multilocus sequence typing (MLST) and characterized their genetic diversity and genetic relationships in order to correlate genotypes/populations with capsular types, resistance profile and clinical symptoms. Moreover, we compared East African camel GBS with East African GBS isolated from humans [16].

## Materials and methods

### Camel GBS isolates used in this study

All work described was in full compliance with national regulations. The work was approved by the ethical committee of the International Livestock Research Institute, which adheres to international standards and is accredited by the National Council of Science and Technology in Kenya (approval number ILRI-IREC2013-12).

Information about the 92 camel GBS isolates used in this study is provided in Additional file 1. The GBS were isolated using standard methods [17] and the species level (GBS) was determined using Lancefield serological grouping as described before [4,5]. Briefly, specimen material was streaked out on Edwards agar plates (Oxoid No. CM0027) containing 5% defibrillated sheep blood. Plates were examined after 24 and 48 h of incubation at 37 °C. Small blue and beta hemolytic colonies were subcultured for further testing. Gram-positive and catalase-negative cocci that were unable to hydrolyze esculin and reacted positive when subjected to Lancefield B testing using Oxoid No. DR587 Latex Grouping Reagent B were considered to be *Streptococcus agalactiae* (GBS).

### Isolation of genomic DNA from camel GBS

The isolates were grown in 10 mL Luria Broth (LB) over night at 37 °C. Culture material was centrifuged at 4 °C and 8000 *g* for 10 min, the supernatant was discarded and the cell pellet was resuspended in 275 μL H_2_O, 10 μL RNAse A (10 mg/mL) and 275 μL TEN buffer (0.05 M Tris, pH 8.0, 0.001 M EDTA, 0.016 M NaCl, 100 mg/mL Lysozyme). The solution was incubated at 37 °C for 30 min, 15 μL 20% SDS and 10 μL proteinase K (20 mg/mL) were added, mixed and the solution was incubated at 37°C for 60 min. 125 μL of 4 M NaCl and 80 μL CTAB solution (10% in H_2_O, preheated to 50°C) were added, mixed and the solution was incubated at 65°C for 10 min. A phenol/chloroform extraction followed by a DNA precipitation using ethanol according to standard protocols [18]. The DNA pellet was resuspended in TE (pH 8.0) buffer and stored at -80 °C for subsequent use.

### In vitro antimicrobial susceptibility testing of camel GBS

The susceptibility of the camel streptococcal isolates to 23 different antimicrobials, listed in Table 1, was assessed phenotypically using broth microdilution method according to CLSI document M31-A3 [19]. Briefly, Sensititre® microtiter plates containing the antimicrobial agents in a vacuum dried form and cation-adjusted Mueller-Hinton Broth supplemented with 2% lysed horse blood (Trek Diagnostic Systems) were used. An inoculation density of 2–8 × 10^5^ CFU/mL was prepared according to CLSI standards. Plates were inoculated using the Sensititre® inoculation system, sealed with a plastic foil and incubated under aerobic conditions at 37 °C for 24 h. We included *Staphylococcus aureus* ATCC 29213 and *Streptococcus pneumonie* ATCC49619 as quality control strains. Since clinical veterinary breakpoints are not available for camels we used the wild type cut-off values for ampicillin, cefoperazone, clindamycin, cefotaxime, erythromycin, tetracycline and vancomycin provided by EUCAST [20] as well as CLSI clinical breakpoints provided for animals [19] (Table 1) for interpretation of results.

**Table 1:** Results of the in vitro antimicrobial susceptibility testing

	**0.008**	**0.015**	**0.03**	**0.06**	**0.12**	**0.25**	**0.5**	**1**	**2**	**4**	**8**	**16**	**32**	**64**	**128**	**ECOFF**	**CLSI CBP**	**MIC**_**90**_
	**mg/L**	**mg/L**	**mg/L**	**mg/L**	**mg/L**	**mg/L**	**mg/L**	**mg/L**	**mg/L**	**mg/L**	**mg/L**	**mg/L**	**mg/L**	**mg/L**	**mg/L**		**(mg/L)**	**(mg/L)**
AMC				2	43	44					1					ND	≥ 32/16	0.25
AMP				1	20	68		1								≤ 0.25	≥ 8	0.25
CEF					1	53	35			1						ND	≥ 32	0.5
CFP					1	44	44		1							≤ 0.125	ND	0.5
CFQ			1	24	65											ND	ND	0.12
CHL								2	83	4	1					ND	≥16	2
CLI			10	80												≤0.5	ND	0.06
CTX			1	52	35	1		1								≤ 0.125	ND	0.12
ENR			1				5	75	8							ND	ND	1
ERY		2	14	74												≤ 0.25	≥ 1	0.06
GEN							1				1	15	71	2		ND	≥ 16	32
OXA		1				4	85									ND	ND	0.5
PEN		1		39	48		1		1							ND	≥4	0.12
PIRL				3	80	7										ND	≥4	0.12
Q-D					1	6	83									ND	ND	0.5
SPI				1		84	4									ND	ND	0.25
SXT			28	57	3	1										ND	≥ 76	0.06
TET						26	31	1					12	19		≤ 1.0	≥ 8	64
TIL						2	1		4	83						ND	ND	4
TUL					1	1	78	10								ND	ND	1
TYL					1	3	56	30								ND	ND	1
VAN							82	8								≤1.0	ND	0.5
XNL				1	5	83		1								ND	≥ 8	0.25

ECOFF-epidemiological cut-off value (retrieved from the European Committee on Antimicrobial Susceptability Testing), CLSI CBP-CLSI M31-A3 veterinary clinical breakpoint for resistance, MIC_90_-Minimum Inhibitory Concentration required to inhibit the growth of 90% of organisms, ND-not determined, GBS-Group B *Streptococcus agalactiae*, AMC-Amoxicillin-clavulanic acid (2:1 ratio), AMP-Ampicillin, CEF – Cephalothin, CFP - Cefoperazone, CFQ – Cefquinome, CHL – Chloramphenicol, CLI – Clindamycin, CTX – Cefotaxime, ENR – Enrofloxacin, ERY – Erythromycin, GEN – Gentamicin, OXA – Oxacillin + 2% NaCl, PEN – Penicillin, PIRL – Pirlimycin, Q-D - Quinupristin/Dalfopristin, SPI – Spiramycin, SXT - Trimethoprim/Sulfamethoxazole, TET – Tetracycline, TIL – Tilmicosin, TUL – Tulathromycin, TYL – Tylosin, VAN – Vancomycin, XNL – Ceftiofur.

### Testing of the presence of the tetracycline resistance genes *tetM*, *tetO,* and Tn*916*-like elements in camel GBS

The presence of the common genes reported to confer resistance to tetracycline i.e. *tetM* and *tetO*, was investigated in camel isolates phenotypically resistant to tetracycline using PCR amplification as described previously [21]. Twenty nanograms of genomic DNA was used as template in a final volume of 50 μL of PCR master mix containing; 1 × PCR buffer (DreamTaq™including MgCl_2_, Fermentas, Germany) 200 μM of dNTP; 600 nM each of the forward/reverse primers and 1.25 U of DreamTaq™ DNA Polymerase. The amplification conditions were as follows; denaturation for 3 min at 95 °C, followed by 35 cycles of 95 °C for 1 min, 48/55 °C for 1 min for *tetM/ tetO* respectively, and 72 °C for 1 min with a final extension of 72 °C for 10 min. Positive controls strains for *tetM* and *tetO* PCRs were *S. agalactiae* 2603 V/R [15] and *Streptococcus anginosus* MG23 [22], respectively. Selected PCR products were sequenced and trimmed from position 865 to 1075 in *tetM* of strain 2603 V/R (GenBank Accession Number: NC_004116). A possible location of the *tetM* gene within a Tn*916*-like element was evaluated using a PCR spanning from the *tetM* gene (*tetM*3-end: 5′-ACTACCGGTGAACCTGTTTG-3′) to the transposase (Tn*916*tnase: 5′-TGGCTCTCTCCAGTCTTTAAG-3′). The expected PCR product was 2,740 bp long. PCR conditions were as outlined above with the difference that the annealing temperature was 58 °C and the extension time was set to 3 min. Positive control strain for Tn*916* was *Staphylococcus rostri* RST11 [23].

### Molecular capsular typing of camel GBS

Capsular polysaccharide gene types (*cps*) were determined using a multiplex PCR assay as described previously [12] using DreamTaq™ DNA Polymerase. Amplified products were separated on a 1.5% agarose gel and the band pattern of the isolates were compared to the band pattern of the GBS reference isolates included in this study representing all known capsular types; Ia, Ib, II, III, IV, V, VI, VII, VIII and IX [12].

### Multilocus Sequence Typing (MLST) of camel GBS

The MLST was performed as described previously [24]. Briefly, seven *S. agalactiae* housekeeping gene loci were amplified, including alcohol dehydrogenase (*adhP*), phenylalanyl tRNA synthetase (*pheS*), amino acid transporter (*atr*), glutamine synthetase (*glnA*), serine dehydratase (*sdhA*), glucose kinase (*glcK*) and transketolase (*tkt*). The PCR amplification was carried out in duplicate (50 μL reaction volume) using GoTaq® Green master mix polymerase (Promega, USA) according to manufacturers’ instructions. PCR products were purified using a QIAquick PCR purification kit (QIAGEN, Germany) and sequenced by Macrogen Inc. (Seoul, Korea). Sequence traces were assembled using the CLC workbench 6. Individual alleles of the camel isolates were compared to the MLST database entries and novel alleles were assigned new allele numbers. New combinations of alleles were also assigned new sequence type (ST) numbers. All camel isolates were submitted to the MLST database [25].

### Minimum Spanning Trees (MST) of camel GBS

To visualize the genetic relationship between camel GBS, MSTs were generated from the allelic profiles of the different isolates using the predefined settings in BioNumerics® 6.6. The clonal complex/populations, capsular type (Ia, Ib, II, III, IV, V, VI, VII, VIII and IX), tetracycline resistance (resistant or susceptible) and clinical complex (chronic cough, mastitis, wound infection/abscess/peri-arthricular abscess, gingivitis, vaginal discharge and healthy camels) were plotted onto the minimum spanning tree using different color codes.

### Phylogenetic analysis of East African GBS isolated from camels and humans

We compared the camel GBS with other published MLST-typed GBS from East Africa [16]. A concatenated sequence of each ST was generated using MLST data from this study and from elsewhere [16]. Jmodeltest 1.0 [26] was used to select the best fitting model of nucleotide substitution, which was found to be the Generalized Time Reversible model with invariant sites and gamma-distributed rate heterogeneity (GTR + I + G) [27]. A maximum likelihood phylogeny for all STs was estimated using this model in PhyML 3.0 [28]. To assess statistical support for the resulting phylogeny, we performed 1000 bootstrap replicates. The tree was drawn using the software FigTree v1.3.1 [29].

### Determination of population structure of East African GBS isolated from camels and humans

For this analysis we included 169 human isolates [16], which were MLST-typed before in order to investigate a possible gene flow between camel and human GBS. The population structure was estimated using the linkage model in STRUCTURE v2.3.2 [30,31]. This Bayesian approach uses multilocus genotypic data to define a set of populations with distinct allele frequencies, and assigns isolates probabilistically to defined populations without prior knowledge of sampling location or sampled host. This program identifies admixtures of isolates and provides an estimate of the percentage ancestry from ancestral population for each isolate. We performed eight replications of the test, in which we initially discarded 10 000 Markov Chain Monte Carlo (MCMC) iterations as *burn-in* and kept the subsequent samples from 30 000 MCMC iterations for analysis. We tested values of *K* between 1 and 10, where *K* is the number of inferred populations. The results of the eight independent runs were averaged for each *K* value to determine the model with the highest likelihood. Real and simulated data have shown that it is not straightforward to determine the optimal value of K when complex population structure is present [30,32] so we also calculated ΔK, a measure of the second order rate of change in the likelihood of K [32] to estimate the appropriate K value for our data.

## Results

### In vitro antimicrobial susceptibility of camel GBS

The susceptibility profiles for 90 out of 92 camel GBS isolates against 23 antimicrobials are presented in Table 1 and Additional file 2. Two strains showed poor growth in Mueller-Hinton Broth with lysed horse blood and were therefore excluded from analysis of minimal inhibitory concentrations (MIC). The interpretation of data is hampered by the absence of clinical breakpoints for camels. We included the epidemiological criterion (wild type cut-offs, ECOFF) for interpretation of data, which unfortunately was only available for a fraction of antimicrobials tested. This criterion was determined on the basis of human strains and a different methodology than the one applied in this study. However, on the basis of veterinary clinical breakpoints available for streptococci [19] and MIC_90_ values determined in this study, only resistance to gentamicin and tetracycline is obvious (Table 1). In fact, 34% of the GBS isolates were resistant to tetracycline. One isolate (ILRI029, ST-617, capsular type VI) showed reduced sensitivity to all β-lactam antibiotics tested, probably due to a mutation in the gene *pbp,* encoding the penicillin binding protein. As expected most GBS isolates showed relatively high MIC to gentamicin in the range of 8–64 mg/L that is characteristic to the genus *Streptococcus*. For all other antimicrobial agents investigated, MIC distributions were normal.

### Prevalence of *tetM*, *tetO* or Tn*916*-like elements

All tetracycline-resistant camel GBS harboured the *tetM* gene. All camel GBS were negative for *tetO*. The 211 bp sequence obtained from the *tetM* amplicon was 100% identical between the isolates investigated (*N* = 17) and 100% identical to other *tetM* sequences in the database from *Staphylococcus aureus* and *S. agalactiae* (e.g. GenBank accession number: NC_004116 and CP003808). In addition, we showed that, in all tetracycline-resistant camel GBS, *tetM* was linked to the Tn*916* transposase, pointing towards a transfer of the resistance gene via a Tn*916*-like element.

### Capsular typing

We assigned a capsular type to all but one camel GBS isolate (ILRI041). The capsular types detected included Ia, II, III, V and VI. The most common types were Ia (37%, 34 isolates), III (27%, 25 isolates) and VI (26%, 24 isolates). Type II and V were less commonly detected with only 4% of the isolates (4 isolates each) being positive for the respective types. Capsular types Ib, IV, VII, VIII and IX were not present among the camel isolates tested.

### Multilocus Sequence Typing (MLST) of camel GBS

Among all 92 camel GBS isolates, fourteen novel alleles were identified by MLST of seven *S. agalactiae* housekeeping gene loci (*adhP*, *pheS, atr*, *glnA*, *sdhA*, *glcK* and *tkt*) [24] (Table 2). All camel isolates differed from GBS currently deposited in the MLST database in alleles of the gene loci *glcK*, *glnA* and *pheS*. A total of 10 new unique sequence types (STs), named ST-609 through ST-618, were identified; the most common STs were ST-617 (29%, 27 isolates), ST-616 (26%, 24 isolates) and ST-612 (26%, 24 isolates). Four isolates belonged to ST-615 and ST-613, respectively, while only two isolates belonged to ST-614, ST-611, ST-610 and ST-609 respectively. Only one isolate represented ST-618 (Additional file 1).

**Table 2:** Camel GBS allelic combinations revealed from MLST typing of 92 East African isolates

**ST**	***adhP***	***pheS***	***atr***	***glnA***	***sdhA***	***glcK***	**tkt**	**Number of isolates**	**% of isolates**
**609**	56	**40**	4	**66**	1	**53**	4	2	2
**610**	13	**40**	6	**65**	3	**52**	**50**	2	2
**611**	13	**40**	**68**	**65**	**54**	**52**	**51**	2	2
**612**	**111**	**40**	6	**65**	3	**52**	**51**	24	26
**613**	**111**	**40**	**67**	**65**	3	**52**	**51**	4	4
**614**	56	**41**	4	**66**	1	**53**	4	2	2
**615**	13	**40**	6	**65**	3	**52**	**51**	4	4
**616**	13	**40**	**68**	**65**	**55**	**52**	4	24	26
**617**	13	**40**	**68**	**65**	3	**52**	**51**	27	29
**618**	**111**	**40**	**69**	**65**	3	**52**	**51**	1	1

Camel specific alleles and sequence types (STs) are displayed in bold.

TUL – Tulathromycin, TYL – Tylosin, VAN – Vancomycin.

### Relationship between camel GBS STs, clonal complexes, capsular types, tetracycline resistance and clinical complexes

**Figure 1:**
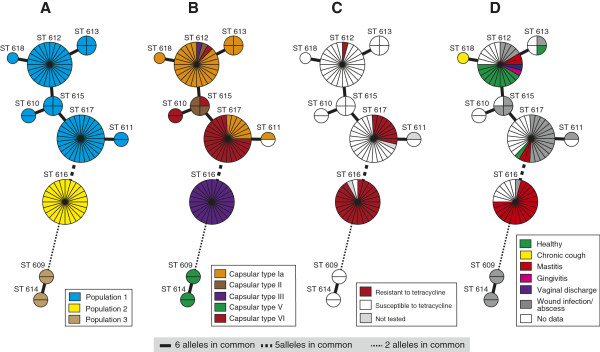
**Minimum spanning tree (MSTree) of East African isolates of camel *S. agalactiae*.** Each circle represents a single sequence type (ST), its size is proportional to the number of isolates. The topological organization within the MSTree is based on a graphical algorithm using an iterative network approach to identify sequential links of increasing distance. **(A)** clonal complex, **(B)** capsular type, **(C)** resistance to tetracycline, and **(D)** clinical symptoms.

According to the minimum spanning tree (MST) network and the number of shared alleles, the ten camel STs clustered in three clonal complexes/populations consisting of ST-609/ST-614, ST-616 and the remaining seven STs that had six alleles in common (Figure 1A). ST-609 and ST-614 were most distantly related to the other camel ST-609 and shared only 2 alleles with ST-616 (Figure 1A).

The ten STs that grouped into 3 clonal complexes were further plotted against capsular type, resistance to tetracycline and clinical complexes. Capsular type Ia was predominant in isolates with ST-618, ST-613 and ST-612, while capsular type VI was most common in isolates with ST-610 and ST-617 (Figure 1B). Isolates with capsular type II were of ST-612 and ST-615. All isolates within ST-616 belonged to capsular type III while all isolates with ST-609/ST-614 belonged to capsular type V (Figure 1B).

Tetracycline resistant isolates were found within ST-612 (4%; 1 out of 24) and ST-617 (30%; 8 out of 26), however the majority of the resistant isolates were grouped within ST-616 (92%; 22 out of 24) representing a total of 71% of all resistant GBS (Figure 1C). Interestingly, most of the GBS isolated from cases of mastitis (81% of mastitis isolates) belonged to ST-616 (Figure 1D). Wound infection and abscesses were found mainly in STs other than ST-616.

No association between STs and geographical origin of the GBS isolates could be detected (data not shown).

### Comparison of camel GBS with other GBS from the region

To investigate the relationship between camel GBS and other GBS from the region, we compared our dataset to previously described human GBS from Kenya [16].

**Figure 2:**
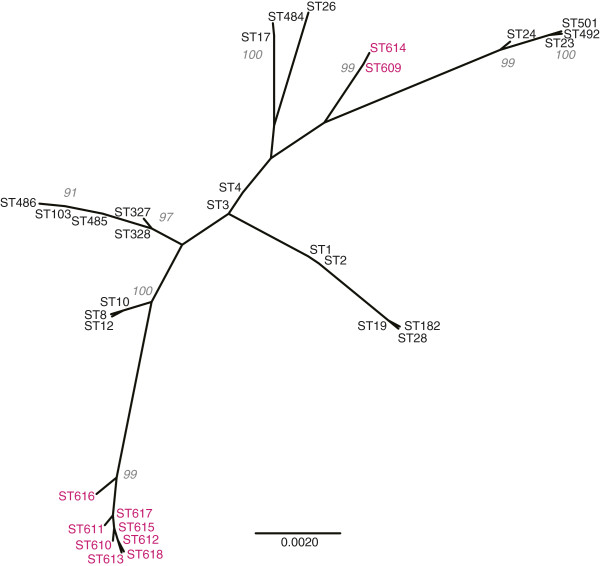
**Unrooted phylogenetic tree displaying the phylogenetic relationship of the East African camel and human *S. agalactiae *isolates.** Camel STs are displayed in colour. The bootstrap values above 90 are displayed.

A maximum likelihood (ML) phylogeny was generated using a total of 33 STs (10 camel GBS STs and 23 human GBS STs [16]). An unrooted tree clearly showed that all camel GBS group in two well supported clusters that are phylogenetically distinct from the Kenyan human GBS (Figure 2). One cluster comprises ST-609/ST-614, the other one all the other isolates.

**Figure 3:**
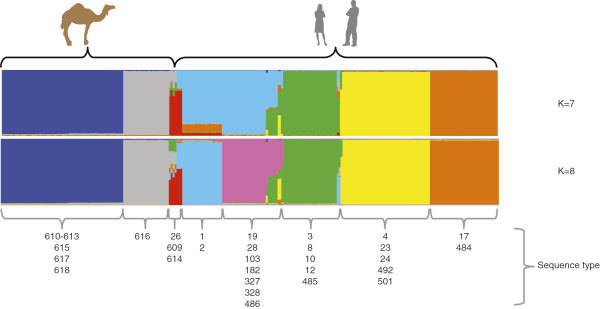
**Population structure of 92 East African camel GBS and 169 Kenyan human GBS.** The populations revealed by the STRUCTURE analysis using the linkage model and sequences from 7 house-keeping gene fragments are displayed below the figure and marked with different colours, the hosts are displayed above. The ancestral parts of each isolate are displayed in vertical lines. The STs are displayed for every population.

In order to look for evidence of genetic exchange between camel and human GBS, we performed a STRUCTURE analysis. Calculation of ΔK produced a modal value for K = 7, whereas the averaged highest likelihood for eight independent runs was obtained for K = 8. We therefore present results for both K = 7 and K = 8 in Figure 3. The seven populations in common for both analyses were; two populations of camel isolates only, corresponding to 2 of the clonal complexes identified on Figure 1A, containing 64 and 24 isolates respectively. All 24 isolates belonging to the second population are of capsular type III, whereas 63 of the 64 isolates belonging to the first population are of capsular type Ia, II, IV or VI. The remaining isolate could not be assigned to a distinct capsular type. Four populations consisted only of human isolates, corresponding to previously defined clonal complexes [16]. The last population consisted of seven isolates, four from camels (ST-609/ST-614) and three from humans, all belonging to capsular type V. These isolates are hybrids with mixed ancestry, the camel isolates having at least 36% shared ancestry with human isolates. The human clonal complexes CC1 and CC19 [16], formed one population for K = 7, but were split into two distinct populations for K = 8.

## Discussion

With a population of many million animals, camels represent a major livestock species in the Horn of Africa. The diet of people living in semiarid and arid regions in the Horn of Africa is to a large extent based on raw camel milk. Therefore, the health status of the camel and specifically of its mammary glands is important for human nutrition in the region. The mapping of capsular types, clinical complexes, and antimicrobial resistance to specific MLST generated sequence types revealed interesting insights into the molecular epidemiology of GBS from camels. We identified three clonal complexes/populations within the camel GBS (Figure 1). The biggest population comprising 64 isolates encompassed four capsular types and isolates from diseased and healthy animals. Abscesses and wound infections accounted for the majority of clinical isolates within this population. The second largest population consisted of 26 isolates, which all belonged to the ST-616 and were of capsular type III only. This population consisted of clinical isolates only, originating from milk of mastitic camels. This population showed the least diversity in terms of capsular types, and clinical complexes and is of most importance to the livestock keeper, since mastitis is the main constraint for productivity of camels. The third population detected in this study contains ST-609 and ST-614. When compared to human isolates, the four camel isolates accounting for STs ST-609 and ST-614 clustered in one population with human GBS isolated in Kenya. The four human isolates belonged to ST-26 and to capsular type V. These isolates represent hybrids of mixed ancestry (Figure 3), pointing towards a high plasticity of *S. agalactiae* and the possible occurrence of genetic exchange [33]. Nevertheless, only three alleles are shared between the camel and human strains within this population, and camel strains are clearly distinct from human strains on the phylogenetic tree (boostrap values on Figure 2). Another study showed that, even if bovine and human strains share all seven alleles of the MLST scheme, they represent distinct lineages, as demonstrated by including more housekeeping genes [34]. Therefore, our data do not provide evidence of cross-species transmission of camel GBS to humans or vice-versa. Nevertheless, GBS from people in intimate contact with camels or camel products should be collected and compared to these strains in order to completely rule out such a possibility.

In order to advise animal holders, caretakers and veterinarians on the best options to treat GBS infections in camels we investigated the susceptibility to 23 anti-microbial drugs used in veterinary and human medicine. Clinical breakpoints which are animal species and disease specific [35] are unfortunately not available for camels. However, the CLSI clinical breakpoints available for streptococci from animal species other than camels [19] as well as the high MIC_90_ values for tetracycline (64 mg/L) and gentamicin (32 mg/L) indicated resistance to these antimicrobials. While the resistance to gentamicin is genus specific and hence expected we additionally detected resistance to tetracycline in 34% of all camel GBS tested. According to EUCAST ECOFF values the MIC_90_ value for tetracycline was high above the cut-off supporting the finding of acquired resistance to tetracycline. Two previous reports based on relatively low numbers of isolates and antimicrobials tested via agar diffusion sensitivity testing reported a higher prevalence of tetracycline resistant isolates (44% to 53%) which might be attributed to the low number of isolates tested, the method used or the sampling scheme [5]. Resistance to tetracycline was detected in three different STs within the two large clonal complexes/populations. Interestingly, not all isolates from any of the three STs were resistant. Most resistant isolates (71%) belonged to the mastitis causing isolates of ST-616. We showed that resistance was conferred by the gene *tetM*. The latter has been reported to be characteristic for resistance against tetracycline especially in human GBS [11]. Interestingly, the presence of the *tetM* gene in three different STs, which represent two distinct populations, suggests a repeated acquisition of the *tetM* gene via transposition by a Tn*916*-like element as indicated by our PCR results [36]. Our current data do not provide a conclusive picture on the source of tetracycline resistance genes detected in camel GBS. Sequence analysis of the flanking regions of *tetM* via full genome sequencing might help in answering this question [37].

Tetracycline is a broad spectrum antimicrobial commonly used to treat bacterial infections in animals in the Horn of Africa. The use of tetracycline and other antimicrobials is not as regulated and closely monitored as in the industrialized world and the entry of antimicrobial residues into the food chain is therefore difficult to control. Our findings show that mastitis in camels caused by GBS should be treated with antimicrobials other than tetracycline to prevent the further spread of tetracycline resistant clones. Alternative drugs are increasingly available in pastoralist regions of East Africa and should be favoured to tetracycline for treatment of mastitis caused by GBS. However, it has to be noted in this respect that one camel GBS isolate of our study revealed an increased MIC to β-lactam antibiotics, assumingly a first step to resistance, indicating that use of this kind of antibiotics in the region might also lead to resistance problems.

Given the increasing importance of camels as dairy animals, and the limitations and risks of parenteral and intra-mammary antibiotic treatments for camel mastitis, long term research into alternative disease control options such as vaccination combined with specific point of care diagnostic tests is highly desirable and timely. In this respect, antigens of capsular type III GBS are a good starting point [38] and require further characterization regarding their potential use in glycogonjugated vaccines or as diagnostics molecules. A vaccine against all camel capsular types would be desirable but is likely to be even more challenging.

Camel GBS should be added to pangenome studies of GBS since they are more distantly related to human strains than livestock species such as cattle given the number of shared alleles [34]. Whole genome sequencing and analysis of camel GBS might reveal supplementary biochemical pathways and functions that are not essential for bacterial survival but which might explain the origin of antibiotic resistance genes or reveal colonization factors necessary to infect the camel. In addition, genome data will allow to identify molecular targets specific to camel GBS for diagnostic tool development.

### Conclusions

Camel GBS sequence types (STs) were distinct from STs reported from other hosts so far. Most mastitis causing GBS were associated with ST-616. Widespread resistance (34%) to tetracycline was most prominent in ST-616 and associated with acquisition of *tetM* carried on a Tn*916*-like element. The presence of *tetM* within different MLST clades suggests acquisition on multiple occasions. Wound infections and mastitis in East African camels associated with GBS should be treated with antimicrobials other than tetracycline in East Africa.

## Abbreviations

GBS: Group B *Streptococcus*; ST: Sequence type; MST: Minimum spanning tree; MLST: Multi locus sequence typing; ECOFF: epidemiological cut-off value.

## Competing interests

The authors declare that they have no competing interests.

## Authors’ contributions

JJ conceived and designed the study. AL, CM, HK, JF, JJ performed the experiments. AF, AL, HK, JJ analysed the data. AF, CAH, CD, EB-R, EdV, HHF, HK, JF, MY, RB contributed reagents/materials/analysis tools. AF, AL, JJ drafted the manuscript. All authors read and approved the final manuscript.

## Supplementary Material

Additional file 1 **Isolates used in this study.** Data on the origin of the isolates tested in this study. Click here for file

Additional file 2 **Minimum inhibitory concentrations of camel *S. agalactiae.*** Minimum inhibitory concentrations were determined on the data available for cattle, GBS-Group B *Streptococcus agalactiae*, AMP-Ampicillin, AMC-Amoxicillin-clavulanic acid, CTX – Cefotaxime, CFP- Cefoperazone, CFQ – Cefquinome, XNL – Ceftiofur, CEF – Cephalothin, CHL – Chloramphenicol, CLI – Clindamycin, ENR – Enrofloxacin, ERY – Erythromycin, GEN – Gentamicin, OXA – Oxacillin, PEN – Penicillin, PIRL – Pirlimycin, Q-D - Quinupristin/Dalfopristin, SPI – Spiramycin, TET – Tetracycline, TIL – Tilmicosin, SXT - Trimethoprim/Sulfamethoxazole. Click here for file
